# Long Non-coding RNA: A Key Regulator in the Pathogenesis of Diabetic Cardiomyopathy

**DOI:** 10.3389/fcvm.2021.655598

**Published:** 2021-04-06

**Authors:** Yaoyao Guo, Xiaohui Feng, Dan Wang, Xiaonan Kang, Lu Zhang, Huihui Ren, Gang Yuan

**Affiliations:** ^1^Department of Endocrinology, Tongji Hospital, Tongji Medical College, Huazhong University of Science and Technology, Wuhan, China; ^2^Branch of National Clinical Research Center for Metabolic Disease, Wuhan, China

**Keywords:** diabetes mellilus, diabetic cardiomyopathy, long non-coding RNA, hypertrophy, fibrosis

## Abstract

In recent years, diabetes mellitus has become a global issue with increasing incidence rate worldwide. Diabetic cardiomyopathy (DCM), one of the important complications of diabetes, refers to patients with type 1 and type 2 diabetes who have ventricular hypertrophy, fibrosis and even diastolic dysfunction. The pathogenesis of DCM is related to oxidative stress, inflammatory response, apoptosis, autophagy, myocardial fibrosis and, diabetic microangiopathy. Long non-coding RNAs (lncRNA) is a non-coding RNA with a length longer than 200 nucleotides which lack the ability of protein coding. With the development of molecular technology, massive evidence demonstrates that lncRNA play a critical role in the molecular mechanism of DCM. Moreover, it can also be used as potential diagnostic markers for DCM. In this review, we intend to summarize the pathological roles and molecular mechanism of lncRNA in the progression of diabetic cardiomyopathy, which may provide promising diagnosis and treatment strategies for DCM.

## Introduction

Diabetes mellitus (DM) is a metabolic syndrome characterized by a high glucose level caused by decreased insulin level or insulin insensitivity. DM currently affects more than 371 million people worldwide and greatly threatens people's lives (1). In patients with DM, diabetic cardiomyopathy (DCM) is a serious diabetic cardiovascular complication, leading to more than half of DM-related mortality cases (2). The main features of DCM are myocardial fibrosis, ventricular enlargement and cardiac dysfunction, which can ultimately result in heart failure (3). The mechanism for the development and progression of DCM is complicated, and local inflammation, mitochondrial dysfunction, oxidative stress, apoptosis, autophagy, and metabolic disturbance have been identified to play a role (4). Recently, emerging genomic technology has also identified a number of non-coding RNAs (ncRNAs) exerting significantly important impact on DCM. Long non-coding RNAs (lncRNAs) are a large cluster of non-protein-coding RNAs longer than 200 nucleotides, but they have the ability to control gene expression in epigenetic, transcriptional, and post-transcriptional levels (5). It is well-established that lncRNAs participate in various biological processes, including cell proliferation and migration, differentiation, inflammation, apoptosis and autophagy (6). Recent studies showed that lncRNAs are aberrantly expressed in diabetic cardiac tissues, and regulating specific lncRNA expression leads to pathophysiological changes of DCM (4). However, the majority of detailed function about lncRNA has not been comprehensively reviewed. Thus, we summarized the relationship between DCM and lncRNAs to further uncover the important roles of lncRNA in the pathogenesis of DCM, and we provided a new perspective about targeting lncRNA as a new therapeutic strategy for a better management of patients with DCM.

## The Definition and Pathogenesis of DCM

DM causes cardiomyopathy and elevates incidence rate of severe cardiovascular events. The term “diabetic cardiomyopathy” was proposed to distinguish the pathophysiological changes observed among these patients from patients with other forms of cardiomyopathy, and it is defined as left ventricular dysfunction that occurs among patients with diabetes mellitus independent of recognized cardiovascular risk factors, such as coronary artery disease or hypertension (7). The main difference between DCM and other cardiomyopathy exists in etiology and pathogenesis. High glucose can induce a series of changes in cardiomyocyte. Impaired mitochondrial and cardiomyocyte calcium handling, myocardial interstitial fibrosis, cardiac autonomic neuropathy and microvascular dysfunction have all been implicated in the development and progression of diabetic cardiomyopathy. However, the occurrence of other cardiomyopathy is related to many factors, such as infection, metabolic disorders, endocrine disease, ischemia, allergy, and other factors (3, 7). DCM was firstly proposed in 1972 by Rubler et al. (8) as a new type of cardiomyopathy in patients with DM, because they found four diabetic patients died of heart failure without history of coronary artery disease, hypertension, or valvular heart diseases. Currently, the most commonly accepted definition of DCM refers to an obvious change in myocardial structure and function, which are observed in patients with diabetes but without other cardiovascular disease, such as uncontrolled hypertension, ischemic disease, coronary artery disease and congenital heart disease (9). Meanwhile, several studies examining biopsies from patients with DCM showed that the pathological feature of DCM includes cardiomyocyte hypertrophy, interstitial fibrosis, PAS-positive material infiltration, coronary arteriole basement membrane thickening and microvascular lesions in the myocardium (10). It has been documented that DCM influences ~12% diabetic patients (11). Clinical studies have described that DCM is one of the leading causes of severe cardiovascular events and worsens the prognosis in diabetic patients (4).

**Figure 1:**
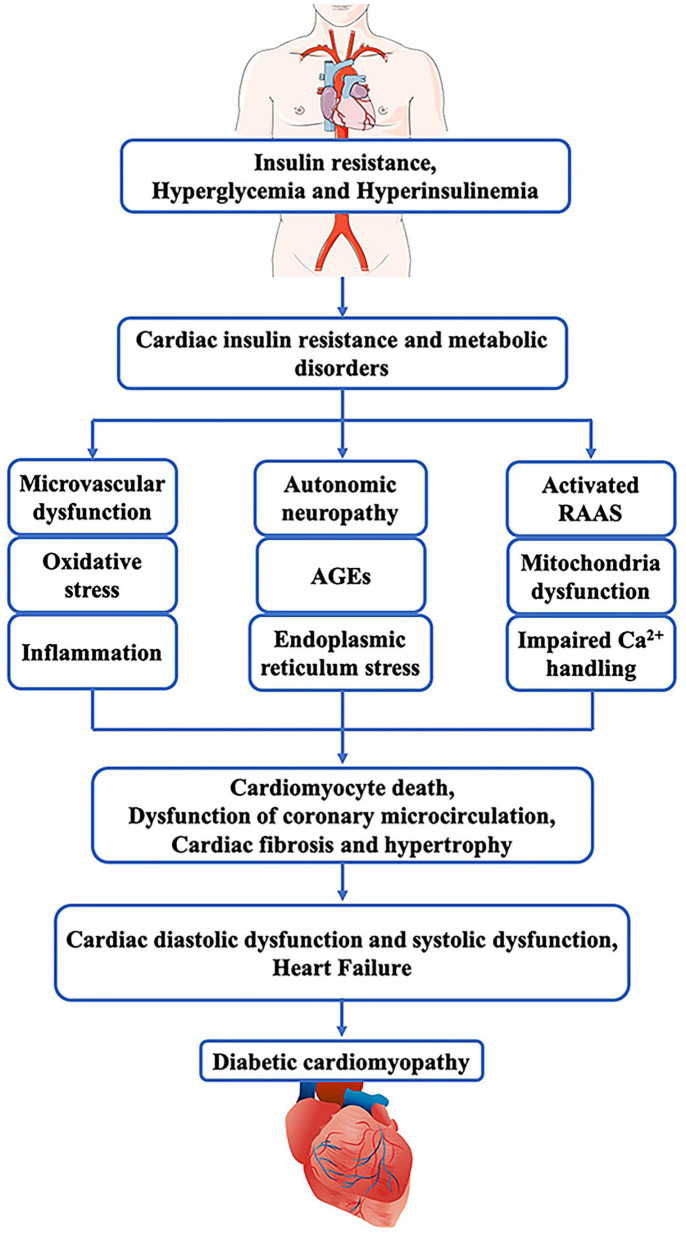
Pathogenesis of diabetic cardiomyopathy. In diabetes mellitus, insulin resistance, hyperglycemia and hyperinsulinemia can cause cardiac insulin resistance and metabolic disorders that induce microvascular dysfunction, Oxidative stress, inflammation, autonomic neuropathy, AGEs, endoplasmic, activated RAAS, mitochondria dysfunction, as well as impaired Ca^2+^ handling. These pathophysiological abnormalities together lead to cardiac fibrosis and hypertrophy, cardiomyocyte death, and dysfunction of coronary microcirculation. Finally, these pathogenic changes in the myocardium impair the diastolic and systolic function of the heart and result in heart failure.

DCM is caused by multiple factors, but evidence for the precise mechanism is still limited. Cardiomyocytes exposing to hyperglycemia condition may develop metabolism disorders that further lead to insulin resistance, mitochondrial dysfunction, upregulation of oxidative stress, inflammatory cytokines, autophagy and apoptosis (11). Hyperglycemia enhances the ingestion and oxidization of fatty acids, which result in the accumulation of toxic lipid intermediates (such as diacylglycerol and ceramide) able to mediate cardiac lipotoxicity (12). Meanwhile, the increased intracellular fatty acids and mitochondrial dysfunction lead to increased production of reactive oxygen species (ROS) and reactive nitrogen species (RNS), which together increase oxidative stress and endoplasmic reticulum (ER) stress and inhibit autophagy (13). Besides, this process is often accompanied by excessive activation of the renin-angiotensin-aldosterone system, which further accelerates cardiac remodeling, including cardiomyocyte hypertrophy, interstitial fibrosis, vascular endothelial cells and vascular smooth muscle cell dysfunction (11). Eventually, the diastolic and systolic function of the myocardium are impaired. In the early stages of diabetes, due to hyperglycemia and insulin resistance, cardiac capillaries can reduce the synthesis of endogenous NO and increase ROS, causing endothelial dysfunction, including inflammation and cell apoptosis (3). Furthermore, in the setting of DM, increased levels of glucose residues and metabolites promote the generation of advanced glycation end products (AGEs), which may also influence cardiomyocytes and endothelial cells (14). Meanwhile, disrupted Ca^2+^ cycling and increased fibrotic scarring in the diabetic heart can mediate contractile arrhythmia and dysfunction, leading to heart failure and death (15). Hence, the main metabolic abnormalities promoting cardiac dysfunction are the progression of hyperglycemia in the heart tissue, compensatory hyperinsulinemia and cardiac insulin resistance, which increase systemic metabolic disorders, activate the SNS and RAAS, prompt mitochondria dysfunction, endoplasmic reticulum stress and oxidative stress and impair Ca2+ homeostasis (16, 17). These effects are associated with dysfunction of coronary microcirculation, cardiomyocyte death, cardiac fibrosis, and hypertrophy. In a word, Insulin resistance and hyperinsulinemia are independently related with the development of diabetic cardiomyopathy and cardiac diastolic and systolic dysfunction (Figure 1). Patients with diabetes mellitus are at high risk of developing diabetic cardiomyopathy (10).

At present, strict glycemic control seems to play a key role in the prevention and treatment of DCM, but new options are still highly demanded to reduce the mortality in diabetic patients. Therefore, further insights into the precise mechanisms behind the advancement of DCM may help facilitate the research and development of clinically effective targets to prevent DCM and its progression to severe cardiovascular events.

## Long Non-Coding RNA

Recent advances in sequencing technologies have demonstrated that RNA, especially the lncRNAs, can act as prime targets for the diagnosis and therapy of specific disease. There has been an explosion in the number of described non-coding genes, which do not encode proteins and show prominent biochemical versatility (18). These ncRNAs can be divided into regulatory ncRNAs and house-keeping ncRNAs or infrastructural ncRNAs. Some of the infrastructural ncRNAs participating in protein targeting can also exert regulatory functions to directly or indirectly control the expression of protein-coding genes (11). Regulatory ncRNAs include long non-coding RNAs (lncRNAs), enhancer RNA (eRNA), microRNA (miRNA), small interference RNA (siRNA), and piwi-interacting RNAs (pi-RNA), all of which may regulate gene expression (19, 20). The diversity of these ncRNAs represents the complexity of these molecules in cell biology and confers regulatory plasticity of gene expression.

**Figure 2:**
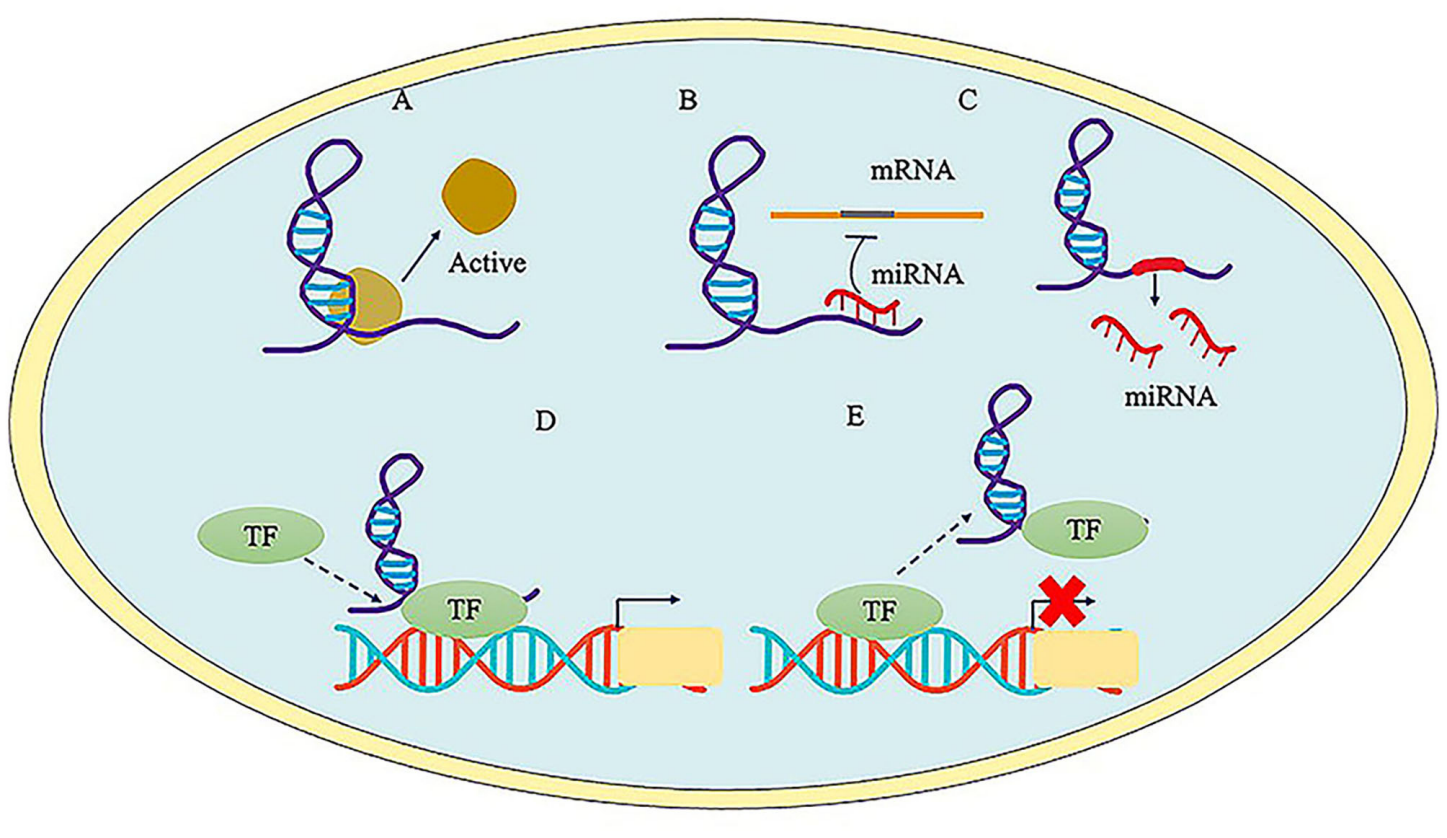
The major function of LncRNAs. **(A)** LncRNA acts as protein scaffolds and regulates protein function. **(B)** LncRNA acts as competitive endogenous RNA to interact with miRNAs. **(C)** LncRNA serves host genes to promote the generation of miRNA. **(D)** LncRNA serves as a guide to promote the transcriptional regulation via tethering transcription factors (TF) to the promoter region. **(E)** LncRNA serves as decoys to inhibit gene expression via interacting with TF.

Most studies estimated that there are ~15,000 lncRNAs in human (21). Long non-coding RNAs are defined as a loosely classified group of long RNAs longer than 200 nucleotides that lack protein-coding abilities (22). Their genes are transcribed by RNA polymerase II (RNAP2), and the transcripts are generally retained in the nuclear, usually but not always undergoing further splicing, 5′-capping and polyadenosine acidification. LncRNAs can be further classified into distinct groups of transcripts according to the genomic location, so these can be intronic, intergenic (large intervening non-coding RNAs or lincRNAs) or antisense to the protein-coding genes (overlapping one or more exons) (11, 21). In addition, these transcripts are associated with multiple proteins and have the ability to exert regulatory effects in multiple biological processes, such as RNA splicing, regulation of chromatin remodeling, nuclear organization, and compartmentalization, or as a competing endogenous RNA (3, 19). The unique function of lncRNAs has been extensively investigated. They can regulate the expression of their neighboring or distant genes through interaction with chromatin complex, DNA, RNA, and proteins (18). Recently, it has been proved that the major functions of lncRNAs at the epigenetic, transcriptional and post-transcriptional levels can be classified into five categories (Figure 2): (1) acting as protein scaffolds to activate or inactivate protein function (23); (2) acting as competing endogenous lncRNA (ceRNA) to interact with miRNA. For example, lncRNA metastasis-associated lung adenocarcinoma transcript 1 (MALAT1) serves as a ceRNA to sponge miR-141 and thus regulates cell growth, proliferation and differentiation in cardiomyocytes (24, 25); (3) acting as host genes to promote the production of miRNAs. For example, lncRNA H19 serve as the reservoir of miR-675-5p and miR-675-3p, which are induced during skeletal muscle differentiation (26); (4) acting as a guide to direct localization of transcription factors to regulate gene expression (27); (5) acting as inhibiting decoys of transcription factors to prevent transcription processes (28, 29). Many studies have demonstrated that overexpression, mutation or ablation of lncRNA genes may affect the progress of numerous human diseases (22).

Currently, more and more investigators focus on the emerging link between lncRNA and diabetes. Following the finding from GWAS, recent transcriptomic analyses revealed that lncRNA expression profiles differed considerably between diabetic cardiomyopathy and other forms of cardiomyopathy (30) LncRNAs have the ability to take part in the development and progression of DCM through multiple pathways, such as gene polymorphism, upregulation or downregulation, methylation, and acetylation. Through lncRNA expression profile analysis of C57BLKS/Jdb/db diabetic mouse model, recent studies indicated that lncRNAs extensively participate in diverse histopathological cardiac changes, such as cardiac fibrosis, cardiomyocyte apoptosis, and autophagy, myocardial hypertrophy and oxidative stress, mainly by regulating the expression of DCM-related miRNAs (11, 31). Studies have identified that lncRNAs are abnormally expressed in diabetic patients and can promote or prevent the occurrence and progression of diabetes, as well as its complications (11). Previous studies have reported that lncRNAs affect cardiac development, homeostasis, and regeneration. Abnormal expression of these lncRNAs is associated with the progression of cardiovascular diseases, and they may serve as diagnostic markers or therapeutic targets (32, 33). However, the data regarding the action mechanism of lncRNAs in DCM are still limited, it is of great significance to study the potential function of lncRNAs in the development of DCM.

## The Regulation of Cardiac-Specific lncRNA in DCM

### H19

H19 is located on human chromosome 11p15.5 and is transcribed downstream the insulin-like growth factor-II (IGF-2) gene (34). H19 is a maternally expressed lncRNA and belongs to a highly conserved imprinted gene cluster, so it has a significant role in cellular proliferation, invasion, apoptosis, and differentiation (35). H19 expression is upregulated during embryogenesis but downregulated after birth, apart from adult skeletal muscle and heart tissue (34). Abnormal gene expression at the H19/IGF2 locus is associated with malignant disorders. For example, H19 can bind to and recruit the histone methyltransferase EZH2, which results in the activation of Wnt/β-catenin and the silencing of the E-cadherin gene in bladder cancer (36). H19 commonly serves as miRNA sponge to sequester miR-106a and the miR-let7 family members. Moreover, H19 is also a precursor of miR-675 to post-translationally regulate all kinds of target genes involved in several cell processes, including growth, proliferation and differentiation (34, 35, 37).

Recently, some studies have revealed a critical role of H19 in the development of DCM. Its upregulation reduces myocardial oxidative stress, cardiomyocyte apoptosis and autophagy in experimental rats, while its downregulation can ameliorate myocardial fibrosis. H19 is an important modulator of cardiomyopathy in diabetic rats. Zhuo et al. (35) found that autophagy-related proteins and autophagosomes are significantly activated in the streptozocin-induced diabetic rat model, and H19 is downregulated in the myocardium. However, after the overexpression of H19, autophagy reaction is reduced, and the left ventricular function is remarkably improved. This is because H19 can restrain autophagy in cardiomyocytes by epigenetically repressing DIRAS3 transcription through interacting with PRC2 (35, 38). Similarly, Li et al. (34) also found that H19 upregulation in diabetic rats attenuates inflammation, oxidative stress and apoptosis, and finally elevates the left ventricular function. They further demonstrated that the H19/miR-675 axis is involved in the modulation of hyperglycemia-induced cardiomyocyte apoptosis by targeting VDAC1 in DCM (34). In contrast, Zhang et al. (37) suggested that H19 knockdown in the myocardium of DCM rats attenuates cardiomyocyte apoptosis, and the H19/miR-675 axis facilitates cardiomyocyte apoptosis by targeting PA2G4. Another pattern for H19 to exert its function is to serve as a competitive endogenous RNA (ceRNA) interacting with protein and microRNA. Huang et al. (39) found that H19 knockout can strengthen the antifibrotic role of miR-455 and attenuate CTGF expression, which further reduces the synthesis of fibrosis-associated protein, such as collagen I, III, and α-SMA. Proteins are another group of partners that are closely linked to H19. H19 can interact with RNA binding proteins, inner membrane protease 1, the Hu family of RNA-binding proteins et al. (40).

In short, lncRNA H19 is associated with DCM, and its upregulation protects myocardial tissue by remission of cardiomyocyte inflammation, oxidative stress, apoptosis, and fibrosis.

### MALAT1

Metastasis-associated lung adenocarcinoma transcript 1 (MALAT1) is an ~8 kb lncRNA located on the short arm of human chromosome 11q13.1 (1). Currently, MALAT1 appears to be greatly relevant to diabetes and its function in DCM development and progression has drawn the attention of many scholars. Downregulation of MALAT1 can reduce cardiomyocyte apoptosis and improve heart function. Zhang et al. (41) reported that MALAT1 is involved in the pathogenesis of DCM. MALAT1 abounds in myocardial tissue, and its expression is considerably upregulated in the myocardial tissue of DM rats. Knock down of MALAT1 by siRNA appreciably reduces inflammatory cytokine concentration (TNF-α, IL-1β, and IL-6) and cell apoptosis, which exerts a positive effect on left ventricular systolic and diastolic function, eventually improving DCM (41). In another study, Bacci et al. (42) indicated that lower nitric oxide (NO) or cGMP bioavailability produced by prolonged DM impairs MALAT1 expression, while sildenafil can reverse the expression of MALAT1 and improve cardiac function by restoring the NO signaling.

MALAT1 can also perform multiple functions by acting as miRNA sponge to sequester miR-141 or miR-181a-5p. Che et al. (24) showed that lncR-MALAT1 serves as ceRNA to prevent the functional availability of miR-141, and overexpression of MALAT1 might enhance the NLRP3 inflammasome activity and TGF-β1/Smads signaling. Thus, melatonin exerts its antifibrotic function caused by its ability to inhibit lncR-MALAT1/miR-141 pathway in DM mice (24). In another study, Cheng et al. (43) demonstrated that MALAT1 expression is upregulated in the myocardium in DCM mice, and knockdown of MALAT1 obviously alleviates hyperglycemia induced cardiomyocyte apoptosis through releasing miR-181a-5p and regulating the p53-p21 pathway. Furthermore, a recent study presents that si-MALAT1 alleviates collagen accumulation and inflammation in hyperglycemia cardiac fibroblasts and DCM mice via the Hippo–YAP pathway and CREB (44). Therefore, knockdown of MALAT1 could serve as a novel therapeutic approach for DCM.

In summary, Malat1 is upregulated in DCM, and MALAT1 knockdown can decrease myocardial fibrosis, inflammation, and apoptosis to improves DCM.

### MIAT

The lncRNA myocardial infarction–associated transcript (MIAT), also known as RNCR2, 2 AK02836, or Gomafu, was first identified to be associated with myocardial infraction in a genome-wide association study in 2006 (45). Since then, more and more evidences have suggested that aberrant MIAT is involved in massive diseases, such as diabetic retinopathy, microvascular dysfunction, myocardial infarction and coronary atherosclerotic heart diseases (31, 46).

In recent years, it is well-documented that MIAT can influence mRNA levels by acting as competing endogenous RNAs (ceRNAs), which can interfere with the miRNA pathways. This model has been proved to be important in DCM (25). For example, Zhou et al. (7) reported that MIAT overexpression can counteract the inhibitory effect of miR-22-3p on death-associated protein kinase 2 (DAPK2), which belongs to a family of calmodulin-dependent serine/threonine kinase and controls cell apoptosis. MIAT knockdown decreases the expression of DAPK2 and inhibits myocardial apoptosis. Thus, MIAT serves as a ceRNA to increase the expression of DAPK2 by sponging miR-22-3p, eventually causing cardiomyocyte apoptosis and subsequent DCM (7). Another study indicated that high glucose-induced MIAT upregulation promotes the production of proinflammatory IL-17 in cardiac muscle tissue. IL-17 is responsible for the pathogenesis of cardiac fibrosis, and MIAT increases IL-17 by attenuating miR214-3p mediated inhibitory effect on IL-17 expression. In addition, MIAT acts as a ceRNA to inhibit the expression of miR-150 in H9c2 cells to promote the development of DCM. Conversely, MIAT knockdown can reduce cardiomyocyte hypertrophy (6, 47). These evidences suggest that the upregulation of MIAT accounts for the pathogenesis of DCM by promoting myocardial apoptosis, fibrosis and hypertrophy.

### GAS5

Growth arrest-specific transcript 5 (GAS5) is a long non-coding RNA (lncRNA) originally isolated from cDNA library in 1988 (48). Gas5 as an important regulatory factor in cell apoptosis and growth is extensively expressed in several tissues and organs in rats and human beings. Recent studies have reported that Gas5 level is associated with the development of cardiovascular disease and DM (49, 50). Tao et al. (48) suggested that GAS5 can function as a ceRNA to regulate the PTEN/MMP-2 signaling pathway by sponging miR-21, which helps to ameliorate cardiac fibrosis. In another study, Zhao et al. (51) described that high glucose leads to Gas5 upregulation in AC16 cells and knockdown of Gas5 prevents AC16 cells from HG-induced injury. Meanwhile, Gas 5 knockdown may alleviate hyperglycemia-induced inflammation partly through suppressing miR-21-5p-mediated TLR4/NF-κB signaling (51). A recent study showed that Gas5 is significantly downregulated in DCM mice, and its overexpression inhibits NLPR3 inflammasome activation-mediated pyroptosis via serving as a ceRNA to enhance AHR expression by squeezing miR-34b-3p (49). Taken together, although the current research about the direct impacts of Gas5 on DCM is insufficient, the cardiac Gas5 could be a valuable target to improve DCM.

### HOTAIR

The HOX transcript antisense RNA (HOTAIR) lncRNA, is a highly abundant and conserved imprinted gene, located at the antisense strand of HOXC gene locus in chromosome 12 (30). Previous studies have proved the function of HOTAIR as a negative regulator of cardiac hypertrophy (CH). HOTAIR is downregulated in both heart tissues from transverse aortic constriction (TAC)-operated mice *in vivo* and cultural cardiomyocytes treated with Ang-II, while its overexpression can attenuates CH through facilitating PTEN expression by competitively inhibiting the function of miR-19 (52). Emerging evidence has highlighted important roles of HOTAIR in the modulation of diabetic cardiomyopathy. For example, Qi and Zhong found that HOTAIR is specifically downregulated in myocardial tissues and serum of DCM patients compared with diabetic patients without cardiomyopathy or healthy controls. Further investigation found that high glucose suppresses HOTAIR expression and Akt phosphorylation, while HOTAIR overexpression activates the PI3K/Akt pathway, which increases the viability of AC16 cells and improves DCM (30). In another study, HOTAIR expression is significantly decreased in diabetic mice hearts, and cardiomyocyte-specific up-regulation of HOTAIR decreases oxidative stress, inflammation, and prevents myocyte from death in mice treated with STZ. The author further proved HOTAIR exerts protective effects in DCM via functioning as a ceRNA to increase SIRT1 expression by sponging miR-34a (53). Therefore, all these findings suggest that HOTAIR may negatively regulate the progress of DCM.

### Crnde

Colorectal Neoplasia Differentially Expressed (Crnde) is a gene locus hCG_1815491 on chromosome 16 and also is also known as a conserved cardiac-specific lncRNA. Crnde has crucial roles in cancer cells, such as promoting proliferation, migration and invasion, and suppressing apoptosis via complicated mechanisms (54). However, a current study show that Crnde expression is negatively correlated with cardiac fibrosis marker genes in an analyze of 376 human heart tissues. Crnde is highly expressed in human and mouse heart tissues, especially cardiac fibroblasts. Zheng et al. (55) found that overexpression of Crnde mitigates cardiac fibrosis and enhances cardiac function in the DCM mouse model, while knockdown of Crnde increases collagen deposition in cardiac fibroblasts. Accordingly, the left ventricular ejection fraction and fractional shortening are reduced. The expression of Crnde is regulated by Smad3, and Crnde reversely inhibits the transcriptional activation of Smad3, forming a Smad3-Crnde negative feedback loop in DCM (55). In a word, Crnde is a potential candidate that protects the heart from diabetes-associated injuries.

### TUG1

Taurine upregulated gene 1 (TUG1), a 7.1-kb lncRNA located at chromosome 22p12, was initially identified as a transcript upregulated by taurine (56). Previous studies reported that TUG1 regulates cell proliferation and migration and is implicated in the development of various cancers, including osteosarcoma, gastric cancer, lung cancer, and other cancers (57). Recent research has reported the function of TUG1 in cardiovascular disease. Li et al. (56) identified that TUG1 is highly expressed in serum samples from atherosclerotic patients, and it can promote the proliferation of atherosclerosis by inhibiting PTEN activity. Similarly, Shi et al. (57) observed that TUG1 is highly expressed in the aorta of spontaneously hypertensive rats and promotes the proliferation and migration of vascular smooth muscle cells via regulating miR-145-5p]. In myocardial infarction, knockdown of TUG1 promotes cell injury under hypoxia through upregulating miR124 (58). Surprisingly, the latest research has found the implication of TUG1 in DCM. The expression of TUG1 is upregulated in cardiomyocytes of db/db mice. *In vivo*, TUG1 knockdown can improve diabetes-induced cardiac hypertrophy and attenuate the fibrosis. In DCM, TUG1 exerts its function possibly by negatively regulating miR-499-5p in cardiomyocytes (59). Therefore, lncRNA TUG1 may be a novel potential target for DCM therapy.

### MEG3

The lncRNA maternally expressed gene 3 (MEG3) is expressed in many human tissues and is located in the imprinted DLK1-MEG3 locus on human chromosome 14q32.3 region (60). Its expression is inhibited in cancer tissue and cells. Gong et al. (61) found that knockdown of MEG3 protects rat cardiomyocyte-derived H9c2 cells from hypoxia-induced injury, Wu et al. (62) discovered a significant upregulation of MEG3 in mouse injured heart after myocardial infarction (61, 62). Recently, Chen et al. (60) found that the expression of MEG3 is upregulated in high glucose-treated AC16 cells, and its knockdown inhibits cardiomyocyte apoptosis. The authors showed that MEG3 acts as a ceRNA for microRNA-145 to induce apoptosis of cardiomyocytes under hyperglycemia conditio]. Thus, MEG3 may represent a promising therapeutic target for the treatment of DCM in the future.

### Kcnq1

The KCNQ1 opposite strand/antisense transcript 1 (Kcnq1ot1) is a 91.5 kb lncRNA located in human chromosome 11p15.5 (63). A growing number of studies have reported that Kcnq1ot1 participates in the progression of several diseases, such as arrhythmia and myocardial damage (64). Currently, Yang et al. (5) displayed that silencing Kcnq1ot1 by a lentivirus-shRNA improves inflammatory necrosis and fibrosis in diabetic myocardial tissue. *In vitro*, Kcnq1ot1 expression is increased in high glucose-treated cardiac fibroblasts, while the left ventricular contraction and diastolic function is deteriorated. Downregulating of Kcnq1ot1 can obviously improve these changes. Further studies have shown that Kcnq1ot1 can serve as the ceRNA to regulate the expression of caspase-1 by sponging miR-214-3p in DCM (5). Therefore, Kcnq1ot1 may potentially be a novel therapeutic target of DCM progression.

### ANRIL

LncRNA ANRIL (a. k. a. CDKN2B-AS1) is located on human chromosome 9 (p21.3) and is transcribed in the opposite direction to the INK4b-ARF-INK4a gene cluster. Thomas et al. (65) proved that ANRIL may interact with epigenetic modifiers at multiple levels in regulating the expression of VEGF and extracellular matrix proteins in the pathogenesis of DCM (65).

### AK081284

AK081284 expression is upregulated in animal models of DCM and myocardial fibrosis, while IL-17 knockdown counteracts the upregulation of AK081284 induced by high glucose. In addition, Zhang et al. (66) reported that enhancing AK081284 expression can promote the production of collagen and transforming growth factor β1 (TGFβ1) in cardiac fibroblasts. Therefore, the IL-17/AK081284/ TGFβ1 pathway plays an important role in high glucose induced collagen accumulation (66).

### DCRF

A recent study by Feng et al. (14) demonstrated that the knockdown of lncRNA DCM-related factor (DCRF) can alleviate myocardial cell autophagy and myocardial fibrosis in diabetic rats. Also, DCRF is upregulated in the myocardium and cardiomyocytes upon high glucose treatment, which induces autophagy. Meanwhile, the authors found that DCRF severs as a ceRNA to reduce the inhibition effect of miR-551b-5p on PCDH17, thereby activating autophagy in cardiomyocytes (14).

### NONRATT007560.2

NONRATT007560.2 (2410nt) was first identified in multiple organs of rats by RNAs sequencing and is located at rat chromosome 12. Recently, Yu et al. (67) found that inhibition of NONATT 007560 expression could reduce ROS accumulation and cardiomyocyte apoptosis after high-glucose stimulation. However, further investigations are needed to uncover the regulatory mechanisms.

### NKILA

Recently, Li et al. (68) performed A 8-year-follow-up study on 312 diabetic patients without complications and found that nuclear factor-κ B interacting long non-coding RNA (lncRNA NKILA) is specifically upregulated in DCM patients, and its knockdown reduces apoptotic cell death in cardiomyocytes under high-glucose treatment (19). Therefore, the inhibition of lncRNA NKILA expression may suppress the development of DCM (68).

### LUCAT1

LncRNA LUCAT1 (lung cancer associated transcript 1) locates on 5q14.3 and has been proved to play a key role in many diseases. Yin et al. (38) showed that the level of LUCAT1 was remarkably upregulated in high glucose-treated AC16 cardiomyocytes, and knockdown of LUCAT1 can improve cardiomyocyte apoptosis and injury by downregulating CYP11B2.

## LncRNA can act as Potential Biomarkers

In current studies, lncRNA is a hotspot in genome-wide differential expression profiling analysis and it has the potential to be a promising candidate and/or target as biomarkers of DCM diagnosis and treatment. For example, Pant et al. (30) performed microarray analysis of lncRNA in T2D mice with and without early DCM, which suggested BC038927, G730013B05Rik, 2700054A10Rik, AK089884, and Daw1 as the core lncRNA with high significance in DCM. Also, Qiu et al. (69) revealed that lncRNA AC061961.2, LING01-AS1, and RP11–13E1.5 were downregulated in myocardial tissues of DCM patients, and downregulation of RP11–557H15.4 and KB-1299A7.2 may also be correlated with DCM progression (69, 70).

In addition, X-inactive specific transcript (XIST) is a functional long non-coding RNA, which has been reported to inhibit the proliferation of cardiomyocytes and promote apoptosis via interacting with miR-130a-3p in myocardial infarction. Chen et al. (12) highlighted lncRNA XIST has the ability to act as an important diagnostic and therapeutic tool against DCM. Meanwhile, subsequent research showed that lncRNA TINCR can also serve as a key factor. It is downregulated in diabetic cardiomyopathy patients.

All these data provide suggests that several lncRNAs may serve as significant diagnostic biomarkers or therapeutic targets to improve the efficacy of diabetic cardiomyopathy diagnosis and therapy in the future.

## Conclusion

In the present review, we summarize the recent progresses about the involvement of lncRNAs in the development of diabetic cardiomyopathy. We show a number of lncRNAs are responsible for the regulation of myocardial fibrosis, cardiomyocyte hypertrophy, oxidative stress, inflammatory response, apoptosis, and autophagy, which are important mechanisms associated with DCM (Table 1). Meanwhile, the various evidence demonstrates that lncRNAs mainly function as ceRNAs to regulate the expression of target genes by sponging miRNAs, and their abnormal expression is closely associated with the pathogenesis of diabetic cardiovascular complications. Currently, although available evidence has indicated that circulating lncRNAs are potential biomarkers for the diagnosis and prognosis of various cardiovascular diseases, more clinical research should be performed to evaluate the diagnostic and prognostic value of lncRNA in DCM. In the future, along with the development of sequencing and CRISPR genome editing technology, it is of great significance to invest more effort to study the involvement of lncRNA in DCM.

**Table 1:** The role of lncRNA in the pathogenesis.

**lncRNA**	**Expression**	**Target genes/miRNA**	**Target cells**	**Pathological mechanism**	**References**
H19	Upregulation	DIRAS3/VDAC1	Cardiomyocytes	Anti-apoptosis	(21, 22)
	Downregulation	miR-455	Cardiomyocytes	Anti-fibrosis	(26)
MALAT1	Downregulation	/	Cardiomyocytes	Anti-apoptosis	(28)
MIAT	Downregulation	miR-22-3p	Cardiomyocytes	Anti-apoptosis	(7)
	Downregulation	miR-214-3p	Cardiomyocytes	Anti-fibrosis	(6)
	Downregulation	miR-150	Cardiomyocytes	Anti-hypertrophy	(37)
GAS5	Downregulation	miR-21-5p	Cardiomyocytes	Anti-inflammatory	(41)
	Upregulation	miR-21	Cardiac fibroblasts	Anti-fibrosis	(38)
	Upregulation	miR-34b-3p	Cardiomyocytes	Anti-pyroptosis	(39)
HOTAIR	Upregulation	Akt	Cardiomyocytes	Cell viability	(19)
	Upregulation	miR-34a	Cardiomyocytes	Anti-oxidative stress and inflammation	(43)
Crnde	Upregulation	Smad3	Cardiac fibroblasts	Anti-cardiac fibrosis	(45)
TUG1	Downregulation	miR-499-5p	Cardiomyocytes	Anti-hypertrophy	(49)
MEG3	Downregulation	miR-145	Cardiomyocytes	Anti-apoptosis	(35)
Kcnq1ot1	Downregulation	miR-214-3p	Cardiomyocytes	Anti-pyroptosis and fibrosis	(5)
ANRIL	Downregulation	P300 and EZH2	Cardiomyocytes	ECM proteins and VEGF	(55)
AK081284	Downregulation	/	Cardiac fibroblasts	Anti-fibrosis	(56)
DCRF	Downregulation	miR-551b-5p	Cardiomyocytes	Anti-autophagy	(13)
NONRATT007560.2	Downregulation	/	Cardiomyocytes	Anti-oxidative stress and apoptosis	(57)
NKILA	Downregulation	/	Cardiomyocytes	Anti-apoptosis	(58)
LUCAT1	Downregulation	/	Cardiomyocytes	Anti-apoptosis	(25)
Neat1	Downregulation	miR-140e5p	Cardiomyocytes	Anti-apoptosis	(61)

## Author Contributions

YG wrote the first draft, which was revised by the remaining authors. All authors made a substantial contribution to conceptualization and writing and agree to be accountable for the content of this review.

## Conflict of Interest

The authors declare that the research was conducted in the absence of any commercial or financial relationships that could be construed as a potential conflict of interest.
